# Biomechanical evaluation of patellar tendon repair using Krackow suture technique

**DOI:** 10.1186/s12938-019-0680-z

**Published:** 2019-05-22

**Authors:** Chen-Yo Yen, Yi-Jung Tsai, Chih-Kun Hsiao, Feng-Chen Kao, Yuan-Kun Tu

**Affiliations:** 1 Department of Orthopedic, E-Da Hospital, No.1, Yi-Da Road, Jiao-Su Village, Yan-Chao District, Kaohsiung City, 824 Taiwan; 2 Department of Medical Research, E-Da Hospital, No.1, Yi-Da Road, Jiao-Su Village, Yan-Chao District, Kaohsiung City, 824 Taiwan; 3 Medical College, I-Shou University, No. 8, Yi-Da Road, Jiao-Su Village, Yan-Chao District, Kaohsiung City, 824 Taiwan

**Keywords:** Patellar tendon rupture, Krackow suture, Cyclic loading, Biomechanics, Tendon repair

## Abstract

**Background:**

Patellar tendon rupture is a potentially devastating injury. Surgical repair is the primary treatment recommended for the patients with patellar tendon ruptures. Given the tendon properties, the suture technique is critical for proper tissue repair. Providing adequate loading during early mobilization is essential to prevent tendon suture repair failure. Therefore, the current study evaluated the mechanical characteristics of various applied loadings on patellar tendon repair using Krackow suture via a porcine model.

**Methods:**

Twelve fresh porcine hindlimbs with patellar tendon rupture were repaired by Krackow method using synthetic and non-absorbable No. 5 Ethibond sutures. Loadings of 100 and 200 N were applied during the cyclic loading test. A three-dimensional optical motion capture system was used to record the gap formation at the initial, 50th, 100th, 150th, 200th, 250th, 500th, 750th, and 1000th cycle. After cyclic loading, the specimen was loaded to failure under displacement control at a rate of 1 mm/s.

**Results:**

Suture breakage was the primary failure mode in both loading conditions. After 1000 cyclic loadings of 100 N, the ultimate failure strength was 243.6 ± 25.8 N. However, the specimens tested under 200 N of loading failed before reaching 200 cycles. Under the 100 N loading, the largest gap deformation (1.89 ± 0.23 mm) and residual deformation (0.213 ± 0.183 mm) were found in the initial cycle. The average cumulative displacement was 5.13 mm from the initial cycle to the 100th cycle and 4.5 mm from the 250th to the 1000th cycle.

**Conclusions:**

Our findings can serve as reference values for further comparisons with various repair techniques or materials. This study suggests that the initially applied load after patellar tendon repair is an important risk factor of re-rupture.

## Background

The patellar tendon is an integral part of the knee extensor mechanism. Although tendon rupture is rare, it is a potentially devastating injury. Approximately, 3–6% of all knee extensor mechanism injuries are patellar tendon ruptures [1]. Rapid contraction of the quadriceps muscle with knee flexion is the primary injury mechanism of patellar tendon rupture, and it often occurs in young athletes involved in jumping activities [2, 3]. The eccentric loading from the quadriceps is transferred to the tendon, and the tendon insertion often absorbs the largest force, and thus, is a common injury site [4]. Other medical problems, such as reduced blood supply, long-term steroid injections, chronic renal disease, or diabetes are risk factors of patellar tendon ruptures. Patients with an age of > 40 years often experience indirect traumas caused by these systemic problems or the administered medications [4, 5].

Surgical repair is the primary treatment recommended for patients with patellar tendon ruptures. Given the tendon properties, the suture technique is critical for proper tissue repair [6, 7]. However, re-rupture rates ranging from 2 to 50% have been reported [8]. Various suture fixation options have been reported to provide adequate suture strength to prevent large gap formation and tissue rupture. The transosseous method with or without cerelage wire for augmentation, Krackow suture, and Bunnell and modified Mason–Allen methods have been used for patellar tendon repair [9–11]. The Krackow method, which is commonly used, has been reported as a locking suture for the fixation of soft tissues, such as ligaments, tendons, or capsular to bone [12]. In the standard repair procedure, two to four transpatellar tunnels are drilled through the patella, and then, the sutures attached to the ruptured tendon can pass though the holes.

Previous studies have reported on the mechanical characteristics of several suture methods [11, 13–15]. The parameters of deformation during cyclic loading and ultimate failure loading are often represented as the biomechanical characteristics of tendon repair. Cyclic loading can simulate functional activities after surgery, and ultimate failure loading can simulate a huge loading event. Krackow et al. reported that loading to failure occurred with approximately 450 N of loading in two rows of Krackow sutures with No. 5 Ethibond sutures [11]. Another study evaluated the standard transpatellar repair technique using the Krackow stitch without augmentation, and the results showed that the displacement after 250 cycles was 11 mm, which was defined as a tendon repair failure [13]. To prevent adhesions and promote performance, post-operative rehabilitation is essential. The long-term follow-up after patellar tendon surgery showed that an early rehabilitation protocol demonstrated good results [16, 17]. Consequently, adequate loading during early mobilization is critical in avoiding tendon repair failure. Therefore, this study aimed to investigate the mechanical characteristics of various applied loadings in patellar tendon repair with Krackow sutures via a porcine model.

## Methods

**Fig. 1:**
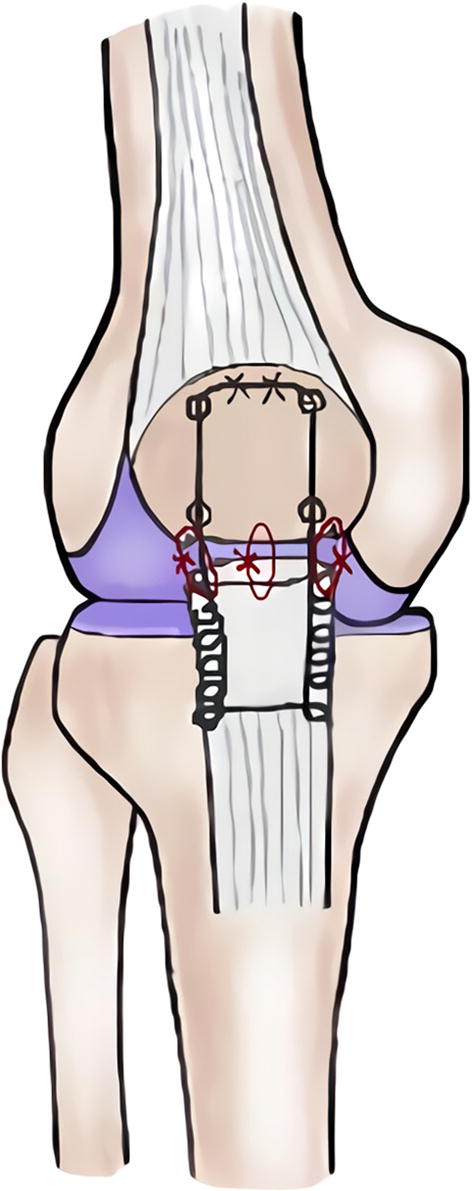
Schematic drawing of patellar tendon repair with Krackow suture method and three single loop augmentation

Twelve fresh porcine hindlimbs were used as the sutured model in the current study; ethical approval was not required. An experienced orthopedic surgeon performed the tendon repair on each specimen. Before the surgery, the soft tissue of each specimen was dissected, and the femur was removed. The rupture was created by the tendon transection that was 3 mm from the edge of the distal pole of the patella. The Krackow method was used with synthetic and non-absorbable No. 5 Ethibond sutures. The suture passed through two longitudinal drill holes in the patella and was tied to the superior pole of the patella to close the tendon rupture gap. Thereafter, a configuration of three single loops was tied to enhance the strength (Fig. 1). Saline solution was used to keep the porcine tendons moist throughout the entire surgical procedure and biomechanical testing.

All the specimens were randomized into two groups with loading levels of 100 and 200 N, respectively. The patella and proximal tibia of each specimen were respectively embedded in two stainless steel cylinders using dental high-strength cement and then installed on the electromechanic testing machine (Instron E3000, USA) for cyclic loading.

LED markers were attached to the patella and tibia to measure the displacement of the tendon. A three-dimensional optical motion capture system (VZ4000, Phoenix Technologies Inc., Canada) was used to collect the relative displacement of the patella and tibia to calculate the displacement of the patellar tendon during cyclic loading testing. The sampling rate of the motion capture system was set at 60 Hz.

The tendon was pretensioned to 20 N within 10 s. Then, the tendons were loaded for 1000 cycles between 20–120 N and 20–220 N at a rate of 1 Hz with sine waves. The gap formations at the initial, 50th, 100th, 150th, 200th, 250th, 500th, 750th, and 1000th cycle were calculated.

After the cycling, each specimen was quasistatically loaded up to failure at a rate of 1 mm/s. Load-to-failure data and the mode of failure were recorded during each experiment. Failure was defined as the maximum force during the test, denoted by a sharp decrease in the loading force, or if the load created a cumulative deformation of 40 mm as observed in the load–displacement curve. Failure mode was defined as rupture of the suture, suture loosening, or fracture of the tendon/bone interface.

## Results

In the 100 N cyclic testing, two specimens experienced failure during the cyclic loading test and four specimens completed the entire testing procedure. However, the specimens in the 200 N cyclic testing all experienced failure before 200 cycles (Table 1). Suture breakage was the primary failure mode during cyclic testing in both loading conditions. After 1000 cycles of loading at 100 N, the ultimate failure strength was 243.6 ± 25.8 N.

**Table 1:** Results of the cyclic loading test with 100 N and 200 N loading

Loading	Cyclic loading test			Load-to-failure test
	No. of failures during cyclic loading	Failure mode/cycle no.		Ultimate strength (*N*)^a^
100 N (*n* = 6)	2	Suture breakage (*n* = 2)	50th–100th cycle (*n* = 2)	243.6 ± 25.8
200 N (*n* = 6)	6	Suture breakage (*n* = 6)	< 10th cycle (*n* = 2) 10th–50th cycle (*n* = 1) 150th–200th cycle (n = 3)	NA

^a^Mean ± standard deviation of four specimens

**Fig. 2:**
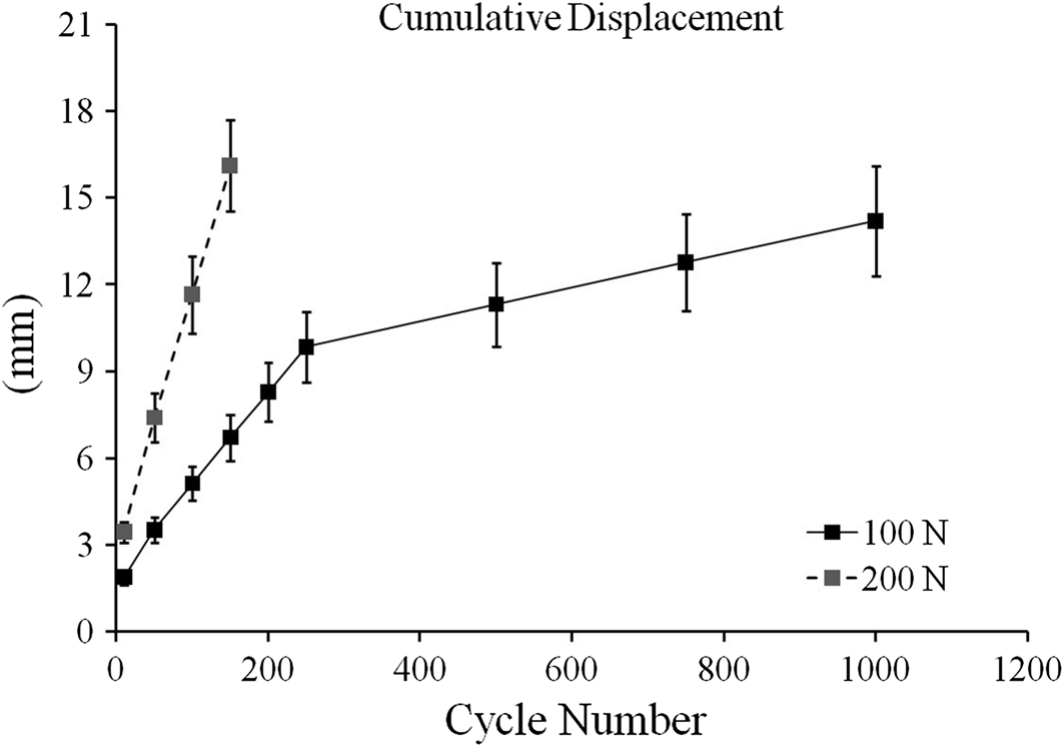
Cumulative displacement at the initial, 50th, 100th, 150th, 200th, 250th, 500th, 750th, and 1000th cycle of 100 N and at the initial, 50th, 100th, and 150th cycle of 200 N

Table 2 lists the average gap deformation and residual deformation at specific cycles. For 100 N, the largest gap deformation and residual deformation were found during the initial cycle. Figure 2 displays the cumulative displacements at the initial, 50th, 100th, 150th, 200th, 250th, 500th, 750th, and 1000th cycle under 100 N and 200 N (from the initial to 150th cycle). The average cumulative displacement from the initial cycle to the 100th cycle was 5.13 and 11.65 mm under 100 and 200 N, respectively. In addition, the cumulative displacement from the 250th to the 1000th cycle was approximately 4.5 mm, which was less than the value before the 250th cycle.

**Table 2:** Gap deformation and residual deformation at specific cycles

	Initial	50th	100th	150th	200th	250th	500th	750th	1000th
Gap deformation (mm)									
100 N^a^	1.89 ± 0.23	1.65 ± 0.17	1.64 ± 0.23	1.61 ± 0.25	1.57 ± 0.23	1.56 ± 0.22	1.50 ± 0.20	1.50 ± 0.22	1.50 ± 0.24
200 N^b^	3.45 ± 0.36	3.96 ± 0.71	4.24 ± 0.48	4.47 ± 0.74	–	–	–	–	–
Residual deformation (mm)									
100 N^a^	0.213 ± 0.183	0.066 ± 0.107	0.017 ± 0.006	0.013 ± 0.008	0.004 ± 0.004	0.007 ± 0.006	0.007 ± 0.004	0.005 ± 0.004	0.011 ± 0.008
200 N^b^	0.228 ± 0.045	0.080 ± 0.040	0.085 ± 0.027	0.042 ± 0.028	–	–	–	–	–

^a^Data collected from four specimens

^b^Data collected from three specimens

## Discussion

The current study discusses the mechanical characteristics of various cyclic loading conditions in a porcine model of patellar tendon suture repair. Cyclic loading tests have been used to represent the regular movements or rehabilitation activities after tendon repair [1, 9, 13, 14]. The parameters of average gap deformation, residual deformation, and cumulative displacement were used to describe the suture results. The gap deformation at a specific cycle represents the suture stability, whereas the residual deformation illustrates the suture loosening in a non-loading condition. The cumulative displacement represents the total loosening of the suture technique, which could be used to compare various repair techniques. In addition, most studies have applied loading under 250 cycles and only reported total elongation length. The elongation and deformation at specific cyclic conditions should be reported because these data could provide reference values and assist in understanding the properties of suture technique, which ultimately will help the surgeons or therapists to set post-operative rehabilitation programs.

During the cyclic loading test, the knot slippage, deformation of the suture, viscoelasticity property, and elastic response of the tendon may contribute to the displacements. Although the deformation at initial cycle was < 5 mm (clinical failure) in our study, the largest gap deformation and residual deformation were found at the initial loading. Early mobilization and rehabilitation following tendon repair have been emphasized [16–18]. An adequate loading during rehabilitation and stronger repair are important to prevent re-rupture or adhesion of the tendon. Previous studies have reported that clinical failure would easily appear (average about 12 loading cycles), and subsequently lead to a lag in knee extensor mechanism repair with suture alone [1, 13]. The tendon repair with augmentation could provide stronger stability to lower the risk of re-rupture and assist the acceleration of rehabilitation program.

Schliemann et al. designed a load-to-failure test and cyclic loading ramp (60 N, 120 N, 180 N, and 240 N) protocol in a porcine model to compare the biomechanical properties of the patellar tendon repair with augmentation [1]. Four different loads were applied for 300 cycles, and a total of 1200 cycles were completed in cyclic testing. The maximum loads reported in load-to-failure testing were approximately 538 N, 445 N, and 344 N for tendon repair with cable wire, polydioxanone suture, and suture anchor, respectively. The total elongation after 1200 cyclic loading was 13.85, 15.40, and 20.09 mm for cable wire, polydioxanone suture, and suture anchor repair, respectively. Although tendon repair with augmentation can tolerate larger loading than Krackow sutures alone at initial cycle loading, applying larger loadings (180 N and 240 N) at the later phase (600th–1200th cycle) still leading to a tendon repair failure. The other study used a bovine model to compare the properties of three repair methods, including #5 Ethibond tendon repair plus wire, #5 FiberWire repair plus augmentation, and #5 Ethibond repair plus #5 FiberWire augmentation [19]. At the static pullout test, the average load at 5 mm gap formation ranged from 115.6 to 91.2 lb, which supports the advantage of augmentation. McKeon et al. compared several configurations of the Krackow stitch in the Achilles tendons of porcines, and no statistical differences were found among two, four, or six locking loops [20]. The mechanical strength of the Krackow suture reported in another study indicated that two single rows of Krackow No. 5 Ethibond sutures showed greater failure strength than the No. 2 Mersiline Bunnell stitch [11]. In addition, the loop configurations of Ethibond sutures have been used in the fixation of patellar fracture. Harrell et al. designed an in vitro study to compare the properties of 18-gauge stainless steel wire, Mersilene and Ethibond, with multiple loops [21]. The findings of their study indicated that the yield strength of multiple No. 5 Ethibond sutures was similar to the strength of 18-gauge stainless steel wire. The results may also support the multiple sutures used in the current study, which could provide better tension in tendon repair. Furthermore, better suture stability may improve the stability of patella, which would prevent the anterior knee pain [22].

Ravalin et al. compared the gap formation after 250 cycles among standard suture repair, suture augmentation, and cable augmentation in a cadaver model [13]. Standard suture repair showed the largest displacement (7.3 mm), whereas suture augmentation and cable augmentation displayed displacements of 4.9 and 3.5 mm, respectively. Another study used the transpatellar method with No. 5 Ethibond sutures and found gap displacements of 3.4, 5.5, 7.3, and 8.5 mm after 1, 10, 100, and 250 cycles, respectively [9]. The cyclic loading tests in both studies simulated the knee movement between 90° of knee flexion and full extension. The loading applied to the tendon with pulley system is different from the bone-tendon-bone animal model. The movement range of the porcine hindlimb is less than that in humans, and the experimental setup in the current study could not represent the actual injury mechanism, which is a limitation of our study.

In the post-operative rehabilitation protocol, isometric exercise, hip muscle strengthening, and passive knee flexion are recommended at the first stage, followed by a gentle active range of motion exercise in the prone position [16, 17]. The average loading during active knee flexion in the prone position is about 70 N, which is less than the loading we used in our study. It would not easily cause re-rupture and could prevent the joint adhesion. However, although the patient could receive early rehabilitation programs and start the range of motion exercise in the prone position, knee flexion in weight-bearing condition should be performed after 6–8 weeks. In our study, all samples that received cyclic loading of 200 N weight failed before 200 cycles, which indicates that weight-bearing exercise, such as slight knee bending, performing during the late phase is recommended to prevent tendon repair failure.

Animal model has been used to investigate the biomechanical performance of knee joint [23, 24]. Although the porcine model we designed could not reflect natural knee movement, specimens from human donors face limitations due to the age of the donor. The donors are usually older than patients who suffer patellar tendon rupture. Furthermore, medications such as steroids or degeneration, which both affect the properties of the patellar tendon, cannot be excluded. Therefore, the porcine model is acceptable in many biomechanical studies. Moreover, the influence of tissue healing after the operation should be considered. In in vivo conditions, the soft tissue may share the loading, and the fixation material or suture may be decreased.

## Conclusion

Our findings can serve as reference values for further comparisons in various repair techniques or materials. This study suggests that the initially applied loads after patellar tendon repair are an important risk factor of re-rupture. More time should be given to the patients before starting the bending exercise under weight-bearing condition to lower the risk of tendon re-rupture.

## Data Availability

The datasets used and analyzed during the current study are available from the corresponding author on reasonable request.
